# *catena*-Poly[[trimethyl­tin(IV)]-μ-(1,1′-binaphthyl-2,2′-diyl phospho­nato)]

**DOI:** 10.1107/S160053680905291X

**Published:** 2009-12-12

**Authors:** Jianjun Wu, Rufen Zhang

**Affiliations:** a College of Chemistry and Chemical Engineering, Liaocheng University, Shandong 252059, People’s Republic of China.

## Abstract

In the title polymeric coordination compound, [Sn(CH_3_)_3_(C_20_H_12_O_4_P)]_*n*_, the Sn atom exhibits a distorted trigonal-bipyramidal coordination geometry with the phosphate O atoms of the 1,1′-binaphthyl-2,2′-diyl phospho­nate ligands in axial positions and equatorial sites occupied by the three methyl groups. Adjacent Sn atoms are bridged by coordination to the two O atoms of each 1,1′-binaphthyl-2,2′-diyl phospho­nate ligand, forming a one-dimensional chain structure parallel to the *b* axis.

## Related literature

For the biological activity of organotin compounds, see: Dubey & Roy (2003 ▶). For related structures, see: Wang *et al.* (2007 ▶); Ma *et al.* (2006 ▶).
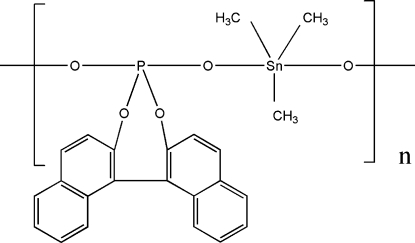


## Experimental

### 

#### Crystal data

[Sn(CH_3_)_3_(C_20_H_12_O_4_P)]
*M* *_r_* = 511.06
Monoclinic, 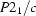

*a* = 18.312 (2) Å
*b* = 10.665 (2) Å
*c* = 11.3361 (18) Å
β = 92.856 (2)°
*V* = 2211.2 (6) Å^3^
*Z* = 4
Mo *K*α radiation
μ = 1.25 mm^−1^
*T* = 298 K
0.42 × 0.21 × 0.13 mm

#### Data collection

Siemens SMART CCD area-detector diffractometer
Absorption correction: multi-scan (*SADABS*; Sheldrick, 1996 ▶) *T*_min_ = 0.621, *T*_max_ = 0.854
11194 measured reflections
3892 independent reflections
2695 reflections with *I* > 2σ(*I*)
*R*_int_ = 0.050

#### Refinement

*R*[*F* ^2^ > 2σ(*F* ^2^)] = 0.036
*wR*(*F* ^2^) = 0.085
*S* = 1.00
3892 reflections
262 parameters
H-atom parameters constrained
Δρ_max_ = 0.76 e Å^−3^
Δρ_min_ = −0.55 e Å^−3^

### 

Data collection: *SMART* (Siemens, 1996 ▶); cell refinement: *SAINT* (Siemens, 1996 ▶); data reduction: *SAINT*; program(s) used to solve structure: *SHELXS97* (Sheldrick, 2008 ▶); program(s) used to refine structure: *SHELXL97* (Sheldrick, 2008 ▶); molecular graphics: *SHELXTL* (Sheldrick, 2008 ▶); software used to prepare material for publication: *SHELXTL*.

## Supplementary Material

Crystal structure: contains datablocks I, global. DOI: 10.1107/S160053680905291X/sj2705sup1.cif

Structure factors: contains datablocks I. DOI: 10.1107/S160053680905291X/sj2705Isup2.hkl

Additional supplementary materials:  crystallographic information; 3D view; checkCIF report

## Figures and Tables

**Table 1:** Selected bond lengths (Å)

Sn1—C21	2.101 (5)
Sn1—C22	2.113 (5)
Sn1—C23	2.123 (5)
Sn1—O3	2.253 (3)
Sn1—O4^i^	2.262 (3)

Symmetry code: (i) 
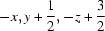
.

**Fig. 1.**
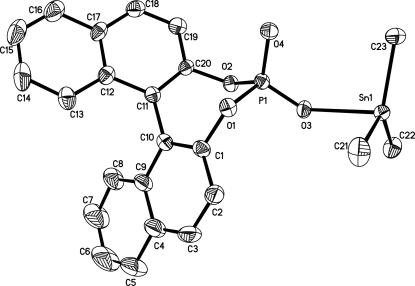
The asymmetric unit of the title compound, showing 30% probability displacement ellipsoids. H atoms have been omitted for clarity.

**Fig. 2.**
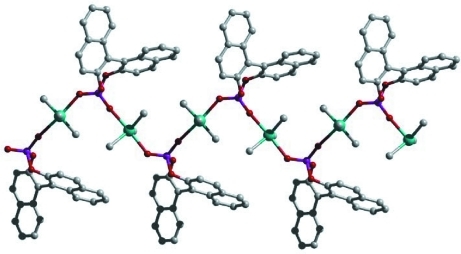
A view of the one-dimensional extended chain structure in the title compound.

## supplementary crystallographic information

### Comment

In recent years, organotin complexes have been attracting more and more attention due to their wide industrial applications and biological activities (Dubey & Roy, 2003). As a part of our ongoing investigations in this field we have synthesized the title compound and present its crystal structure here. The title compound, which is shown in Fig.1, forms an extended one-dimensional chain structure arising from Sn—O bridges formed by the 1,1'-binaphthyl-2,2'-diyl phosphonate ligands. The Sn—O bond distances in the compound (Sn(1)—O(3) = 2.253 (3) Å; Sn(1)—O(4)#1 = 2.262 (3) Å; symmetry code (#1): #1 - *x*,*y* + 1/2,-*z* + 3/2) are comparable to those found in related organotin carboxylates (Ma *et al.* 2006, Wang *et al.*  2007). The Sn atom assumes a slightly distorted trigonal-bipyramidal coordination geometry, provided by and three methyl groups in the equatorial positions and two O atoms of symmetry related phosphate groups in the axial positions.

### Experimental

1,1'-Binaphthyl-2,2'-diyl phosphonate acid (1 mmol) and sodium ethoxide (1.2 mmol) were added to a stirred solution of benzene (30 ml) in a Schlenk flask and stirred for 0.5 h under nitrogen. Trimethyltin chloride (1 mmol) was then added to the reactor and the reaction mixture was stirred for 12 h at room temperature. The resulting clear solution was evaporated under vacuum. The product was crystallized from a solution of diethyl ether to yield colourless blocks of the title compound (yield 83%). Anal. Calcd (%) for C_23_H_21_O_4_P_1_Sn_1_ (Mr = 511.06): C, 54.05; H, 4.14. Found (%): C, 54.51; H, 4.64.

### Refinement

The H atoms were positioned geometrically, with methyl C—H distances of 0.96Å and aromatic C—H distances of 0.93 Å, and refined as riding on their parent atoms, with *U*_iso_(H) = 1.2 *U*_eq_(C_aromatic_) or 1.5 *U*_eq_(C) for the methyl groups.

### Crystal data

**Table:**

[Sn(CH_3_)_3_(C_20_H_12_O_4_P)]	*F*(000) = 1024
*M**_r_* = 511.06	*D*_x_ = 1.535 Mg m^−^^3^
Monoclinic, *P*2_1_/*c*	Mo *K*α radiation, λ = 0.71073 Å
Hall symbol: -P 2ybc	Cell parameters from 3010 reflections
*a* = 18.312 (2) Å	θ = 2.6–25.4°
*b* = 10.665 (2) Å	µ = 1.25 mm^−^^1^
*c* = 11.3361 (18) Å	*T* = 298 K
β = 92.856 (2)°	Plate, colorless
*V* = 2211.2 (6) Å^3^	0.42 × 0.21 × 0.13 mm
*Z* = 4	

### Data collection

**Table:**

Siemens SMART CCD area-detector diffractometer	3892 independent reflections
Radiation source: fine-focus sealed tube	2695 reflections with *I* > 2σ(*I*)
graphite	*R*_int_ = 0.050
φ and ω scans	θ_max_ = 25.0°, θ_min_ = 2.2°
Absorption correction: multi-scan (*SADABS*; Sheldrick, 1996)	*h* = −21→19
*T*_min_ = 0.621, *T*_max_ = 0.854	*k* = −11→12
11194 measured reflections	*l* = −12→13

### Refinement

**Table:**

Refinement on *F*^2^	Primary atom site location: structure-invariant direct methods
Least-squares matrix: full	Secondary atom site location: difference Fourier map
*R*[*F*^2^ > 2σ(*F*^2^)] = 0.036	Hydrogen site location: inferred from neighbouring sites
*wR*(*F*^2^) = 0.085	H-atom parameters constrained
*S* = 1.00	*w* = 1/[σ^2^(*F*_o_^2^) + (0.0333*P*)^2^ + 1.120*P*] where *P* = (*F*_o_^2^ + 2*F*_c_^2^)/3
3892 reflections	(Δ/σ)_max_ = 0.001
262 parameters	Δρ_max_ = 0.76 e Å^−^^3^
0 restraints	Δρ_min_ = −0.55 e Å^−^^3^

### Fractional atomic coordinates and isotropic or equivalent isotropic displacement parameters (Å^2^)

**Table:**

	*x*	*y*	*z*	*U*_iso_*/*U*_eq_	
Sn1	0.023494 (17)	0.37358 (3)	0.73733 (3)	0.03206 (12)	
O1	0.16136 (16)	0.0701 (3)	0.8547 (3)	0.0387 (8)	
O2	0.18645 (16)	0.0599 (3)	0.6397 (3)	0.0366 (8)	
O3	0.11465 (16)	0.2375 (3)	0.7063 (3)	0.0381 (8)	
O4	0.05909 (17)	0.0202 (3)	0.7143 (3)	0.0447 (9)	
P1	0.12454 (6)	0.10124 (12)	0.72665 (11)	0.0332 (3)	
C1	0.2357 (3)	0.0974 (5)	0.8797 (4)	0.0412 (13)	
C2	0.2521 (3)	0.1921 (5)	0.9613 (5)	0.0558 (16)	
H2	0.2151	0.2343	0.9982	0.067*	
C3	0.3238 (4)	0.2212 (6)	0.9858 (6)	0.074 (2)	
H3	0.3357	0.2830	1.0414	0.088*	
C4	0.3800 (4)	0.1599 (7)	0.9291 (6)	0.0705 (19)	
C5	0.4550 (5)	0.1966 (8)	0.9479 (8)	0.106 (3)	
H5	0.4676	0.2599	1.0016	0.127*	
C6	0.5076 (4)	0.1390 (10)	0.8872 (10)	0.123 (4)	
H6	0.5562	0.1634	0.8997	0.147*	
C7	0.4903 (4)	0.0451 (9)	0.8073 (8)	0.105 (3)	
H7	0.5273	0.0079	0.7663	0.126*	
C8	0.4195 (3)	0.0051 (7)	0.7868 (6)	0.074 (2)	
H8	0.4089	−0.0587	0.7327	0.088*	
C9	0.3627 (3)	0.0616 (6)	0.8487 (5)	0.0541 (16)	
C10	0.2878 (3)	0.0255 (5)	0.8278 (4)	0.0397 (13)	
C11	0.2655 (2)	−0.0811 (5)	0.7489 (4)	0.0364 (12)	
C12	0.2897 (3)	−0.2077 (5)	0.7690 (5)	0.0438 (13)	
C13	0.3342 (3)	−0.2435 (6)	0.8696 (5)	0.0557 (16)	
H13	0.3511	−0.1826	0.9230	0.067*	
C14	0.3524 (3)	−0.3664 (7)	0.8891 (7)	0.077 (2)	
H14	0.3805	−0.3886	0.9564	0.092*	
C15	0.3290 (4)	−0.4585 (7)	0.8084 (8)	0.080 (2)	
H15	0.3423	−0.5416	0.8219	0.096*	
C16	0.2871 (3)	−0.4285 (6)	0.7108 (7)	0.0682 (19)	
H16	0.2722	−0.4909	0.6576	0.082*	
C17	0.2659 (3)	−0.3027 (5)	0.6891 (5)	0.0471 (14)	
C18	0.2189 (3)	−0.2722 (5)	0.5902 (5)	0.0504 (15)	
H18	0.2054	−0.3339	0.5354	0.060*	
C19	0.1937 (3)	−0.1536 (5)	0.5750 (5)	0.0459 (14)	
H19	0.1618	−0.1342	0.5112	0.055*	
C20	0.2158 (2)	−0.0606 (5)	0.6556 (4)	0.0353 (12)	
C21	0.0532 (3)	0.3602 (6)	0.9183 (4)	0.0700 (19)	
H21A	0.1034	0.3844	0.9315	0.105*	
H21B	0.0470	0.2754	0.9442	0.105*	
H21C	0.0228	0.4148	0.9620	0.105*	
C22	0.0762 (3)	0.5007 (5)	0.6267 (5)	0.0535 (15)	
H22A	0.1202	0.5308	0.6666	0.080*	
H22B	0.0443	0.5701	0.6081	0.080*	
H22C	0.0881	0.4589	0.5551	0.080*	
C23	−0.0628 (3)	0.2641 (5)	0.6592 (5)	0.0515 (15)	
H23A	−0.0445	0.1828	0.6393	0.077*	
H23B	−0.0821	0.3049	0.5889	0.077*	
H23C	−0.1007	0.2550	0.7140	0.077*	

### Atomic displacement parameters (Å^2^)

**Table:**

	*U*^11^	*U*^22^	*U*^33^	*U*^12^	*U*^13^	*U*^23^
Sn1	0.0352 (2)	0.0324 (2)	0.02855 (18)	−0.00087 (18)	0.00089 (13)	0.00060 (16)
O1	0.038 (2)	0.045 (2)	0.0332 (19)	0.0040 (16)	0.0052 (15)	−0.0005 (16)
O2	0.0348 (19)	0.039 (2)	0.0361 (19)	0.0029 (16)	0.0061 (15)	−0.0013 (16)
O3	0.0366 (19)	0.034 (2)	0.044 (2)	0.0063 (16)	0.0054 (15)	0.0004 (16)
O4	0.0342 (19)	0.040 (2)	0.060 (2)	−0.0055 (17)	0.0056 (16)	0.0063 (18)
P1	0.0302 (7)	0.0349 (9)	0.0347 (7)	0.0007 (6)	0.0047 (5)	0.0000 (6)
C1	0.044 (3)	0.045 (4)	0.034 (3)	−0.003 (3)	−0.008 (2)	0.004 (2)
C2	0.071 (4)	0.047 (4)	0.048 (4)	0.008 (3)	−0.014 (3)	−0.006 (3)
C3	0.093 (5)	0.056 (5)	0.069 (4)	−0.011 (4)	−0.035 (4)	−0.010 (4)
C4	0.057 (4)	0.068 (5)	0.084 (5)	−0.015 (4)	−0.022 (4)	0.009 (4)
C5	0.078 (6)	0.101 (7)	0.133 (8)	−0.030 (5)	−0.041 (6)	−0.006 (6)
C6	0.050 (5)	0.144 (10)	0.170 (10)	−0.035 (6)	−0.023 (6)	0.021 (8)
C7	0.048 (5)	0.134 (9)	0.134 (8)	−0.013 (5)	0.000 (5)	0.009 (7)
C8	0.037 (4)	0.086 (6)	0.098 (5)	−0.004 (4)	0.003 (3)	0.013 (4)
C9	0.040 (3)	0.058 (4)	0.063 (4)	−0.006 (3)	−0.010 (3)	0.005 (3)
C10	0.033 (3)	0.045 (3)	0.040 (3)	0.000 (2)	−0.001 (2)	0.002 (3)
C11	0.025 (3)	0.041 (3)	0.044 (3)	0.002 (2)	0.010 (2)	−0.005 (2)
C12	0.038 (3)	0.041 (4)	0.054 (4)	0.005 (3)	0.015 (3)	−0.001 (3)
C13	0.045 (3)	0.062 (4)	0.060 (4)	0.013 (3)	0.008 (3)	0.008 (3)
C14	0.065 (4)	0.075 (6)	0.091 (5)	0.029 (4)	0.011 (4)	0.026 (5)
C15	0.068 (5)	0.050 (5)	0.124 (7)	0.017 (4)	0.028 (5)	0.016 (5)
C16	0.057 (4)	0.049 (4)	0.100 (6)	0.006 (3)	0.021 (4)	−0.007 (4)
C17	0.040 (3)	0.034 (3)	0.069 (4)	0.006 (3)	0.023 (3)	0.000 (3)
C18	0.044 (3)	0.046 (4)	0.062 (4)	−0.002 (3)	0.013 (3)	−0.015 (3)
C19	0.041 (3)	0.053 (4)	0.045 (3)	0.000 (3)	0.009 (2)	−0.012 (3)
C20	0.032 (3)	0.032 (3)	0.043 (3)	0.001 (2)	0.010 (2)	−0.004 (2)
C21	0.095 (5)	0.091 (5)	0.022 (3)	0.034 (4)	−0.006 (3)	−0.004 (3)
C22	0.057 (4)	0.042 (4)	0.063 (4)	−0.002 (3)	0.022 (3)	0.012 (3)
C23	0.051 (3)	0.045 (4)	0.058 (4)	−0.007 (3)	−0.006 (3)	−0.006 (3)

### Geometric parameters (Å, °)

**Table:**

Sn1—C21	2.101 (5)	C9—C10	1.432 (7)
Sn1—C22	2.113 (5)	C10—C11	1.491 (7)
Sn1—C23	2.123 (5)	C11—C20	1.378 (6)
Sn1—O3	2.253 (3)	C11—C12	1.436 (7)
Sn1—O4^i^	2.262 (3)	C12—C17	1.414 (7)
O1—C1	1.407 (5)	C12—C13	1.421 (7)
O1—P1	1.605 (3)	C13—C14	1.367 (8)
O2—C20	1.401 (6)	C13—H13	0.9300
O2—P1	1.601 (3)	C14—C15	1.396 (9)
O3—P1	1.481 (3)	C14—H14	0.9300
O4—P1	1.479 (3)	C15—C16	1.352 (9)
O4—Sn1^ii^	2.262 (3)	C15—H15	0.9300
C1—C10	1.379 (7)	C16—C17	1.415 (8)
C1—C2	1.392 (7)	C16—H16	0.9300
C2—C3	1.366 (8)	C17—C18	1.416 (7)
C2—H2	0.9300	C18—C19	1.356 (7)
C3—C4	1.402 (9)	C18—H18	0.9300
C3—H3	0.9300	C19—C20	1.394 (7)
C4—C9	1.415 (9)	C19—H19	0.9300
C4—C5	1.434 (9)	C21—H21A	0.9600
C5—C6	1.358 (12)	C21—H21B	0.9600
C5—H5	0.9300	C21—H21C	0.9600
C6—C7	1.377 (12)	C22—H22A	0.9600
C6—H6	0.9300	C22—H22B	0.9600
C7—C8	1.373 (9)	C22—H22C	0.9600
C7—H7	0.9300	C23—H23A	0.9600
C8—C9	1.418 (8)	C23—H23B	0.9600
C8—H8	0.9300	C23—H23C	0.9600
			
C21—Sn1—C22	121.3 (2)	C9—C10—C11	122.4 (5)
C21—Sn1—C23	121.4 (2)	C20—C11—C12	117.2 (5)
C22—Sn1—C23	117.3 (2)	C20—C11—C10	119.4 (5)
C21—Sn1—O3	87.19 (17)	C12—C11—C10	123.3 (5)
C22—Sn1—O3	87.53 (17)	C17—C12—C13	117.9 (5)
C23—Sn1—O3	97.01 (17)	C17—C12—C11	119.3 (5)
C21—Sn1—O4^i^	87.34 (18)	C13—C12—C11	122.7 (5)
C22—Sn1—O4^i^	91.78 (17)	C14—C13—C12	120.9 (6)
C23—Sn1—O4^i^	89.37 (17)	C14—C13—H13	119.6
O3—Sn1—O4^i^	173.14 (12)	C12—C13—H13	119.6
C1—O1—P1	119.9 (3)	C13—C14—C15	120.3 (7)
C20—O2—P1	116.9 (3)	C13—C14—H14	119.8
P1—O3—Sn1	133.94 (18)	C15—C14—H14	119.8
P1—O4—Sn1^ii^	158.4 (2)	C16—C15—C14	120.8 (7)
O4—P1—O3	117.80 (19)	C16—C15—H15	119.6
O4—P1—O2	112.09 (19)	C14—C15—H15	119.6
O3—P1—O2	105.04 (18)	C15—C16—C17	120.4 (7)
O4—P1—O1	105.49 (19)	C15—C16—H16	119.8
O3—P1—O1	112.72 (19)	C17—C16—H16	119.8
O2—P1—O1	102.81 (17)	C12—C17—C16	119.7 (6)
C10—C1—C2	123.8 (5)	C12—C17—C18	119.9 (5)
C10—C1—O1	118.9 (4)	C16—C17—C18	120.4 (6)
C2—C1—O1	117.2 (5)	C19—C18—C17	120.3 (5)
C3—C2—C1	118.2 (6)	C19—C18—H18	119.8
C3—C2—H2	120.9	C17—C18—H18	119.8
C1—C2—H2	120.9	C18—C19—C20	119.6 (5)
C2—C3—C4	121.4 (6)	C18—C19—H19	120.2
C2—C3—H3	119.3	C20—C19—H19	120.2
C4—C3—H3	119.3	C11—C20—C19	123.3 (5)
C3—C4—C9	119.7 (6)	C11—C20—O2	118.6 (4)
C3—C4—C5	121.6 (7)	C19—C20—O2	118.1 (4)
C9—C4—C5	118.7 (7)	Sn1—C21—H21A	109.5
C6—C5—C4	120.0 (8)	Sn1—C21—H21B	109.5
C6—C5—H5	120.0	H21A—C21—H21B	109.5
C4—C5—H5	120.0	Sn1—C21—H21C	109.5
C5—C6—C7	121.1 (8)	H21A—C21—H21C	109.5
C5—C6—H6	119.5	H21B—C21—H21C	109.5
C7—C6—H6	119.5	Sn1—C22—H22A	109.5
C8—C7—C6	121.5 (8)	Sn1—C22—H22B	109.5
C8—C7—H7	119.3	H22A—C22—H22B	109.5
C6—C7—H7	119.3	Sn1—C22—H22C	109.5
C7—C8—C9	119.6 (7)	H22A—C22—H22C	109.5
C7—C8—H8	120.2	H22B—C22—H22C	109.5
C9—C8—H8	120.2	Sn1—C23—H23A	109.5
C4—C9—C8	119.2 (6)	Sn1—C23—H23B	109.5
C4—C9—C10	119.2 (6)	H23A—C23—H23B	109.5
C8—C9—C10	121.6 (6)	Sn1—C23—H23C	109.5
C1—C10—C9	117.2 (5)	H23A—C23—H23C	109.5
C1—C10—C11	120.3 (4)	H23B—C23—H23C	109.5
			
C21—Sn1—O3—P1	73.2 (3)	C2—C1—C10—C11	174.8 (5)
C22—Sn1—O3—P1	−165.3 (3)	O1—C1—C10—C11	−1.5 (7)
C23—Sn1—O3—P1	−48.1 (3)	C4—C9—C10—C1	5.8 (8)
O4^i^—Sn1—O3—P1	110.3 (10)	C8—C9—C10—C1	−171.2 (5)
Sn1^ii^—O4—P1—O3	−128.8 (5)	C4—C9—C10—C11	−177.0 (5)
Sn1^ii^—O4—P1—O2	109.1 (6)	C8—C9—C10—C11	6.0 (8)
Sn1^ii^—O4—P1—O1	−2.0 (6)	C1—C10—C11—C20	53.0 (7)
Sn1—O3—P1—O4	32.8 (4)	C9—C10—C11—C20	−124.1 (5)
Sn1—O3—P1—O2	158.4 (2)	C1—C10—C11—C12	−123.1 (5)
Sn1—O3—P1—O1	−90.5 (3)	C9—C10—C11—C12	59.9 (7)
C20—O2—P1—O4	−61.8 (4)	C20—C11—C12—C17	4.1 (7)
C20—O2—P1—O3	169.1 (3)	C10—C11—C12—C17	−179.8 (4)
C20—O2—P1—O1	51.0 (3)	C20—C11—C12—C13	−172.6 (4)
C1—O1—P1—O4	158.7 (4)	C10—C11—C12—C13	3.5 (7)
C1—O1—P1—O3	−71.5 (4)	C17—C12—C13—C14	−0.7 (8)
C1—O1—P1—O2	41.1 (4)	C11—C12—C13—C14	176.0 (5)
P1—O1—C1—C10	−71.3 (5)	C12—C13—C14—C15	1.6 (9)
P1—O1—C1—C2	112.1 (5)	C13—C14—C15—C16	−1.0 (10)
C10—C1—C2—C3	4.5 (9)	C14—C15—C16—C17	−0.4 (10)
O1—C1—C2—C3	−179.2 (5)	C13—C12—C17—C16	−0.6 (7)
C1—C2—C3—C4	1.3 (9)	C11—C12—C17—C16	−177.5 (5)
C2—C3—C4—C9	−3.0 (10)	C13—C12—C17—C18	177.3 (5)
C2—C3—C4—C5	175.2 (6)	C11—C12—C17—C18	0.5 (7)
C3—C4—C5—C6	−176.9 (8)	C15—C16—C17—C12	1.2 (8)
C9—C4—C5—C6	1.4 (12)	C15—C16—C17—C18	−176.7 (5)
C4—C5—C6—C7	−0.1 (15)	C12—C17—C18—C19	−3.5 (8)
C5—C6—C7—C8	−0.7 (15)	C16—C17—C18—C19	174.4 (5)
C6—C7—C8—C9	0.2 (12)	C17—C18—C19—C20	1.8 (8)
C3—C4—C9—C8	176.5 (6)	C12—C11—C20—C19	−6.1 (7)
C5—C4—C9—C8	−1.9 (9)	C10—C11—C20—C19	177.7 (4)
C3—C4—C9—C10	−0.6 (9)	C12—C11—C20—O2	174.8 (4)
C5—C4—C9—C10	−179.0 (6)	C10—C11—C20—O2	−1.5 (6)
C7—C8—C9—C4	1.1 (10)	C18—C19—C20—C11	3.2 (7)
C7—C8—C9—C10	178.1 (6)	C18—C19—C20—O2	−177.7 (4)
C2—C1—C10—C9	−8.0 (8)	P1—O2—C20—C11	−76.4 (5)
O1—C1—C10—C9	175.7 (4)	P1—O2—C20—C19	104.4 (4)

Symmetry codes: (i) −*x*, *y*+1/2, −*z*+3/2; (ii) −*x*, *y*−1/2, −*z*+3/2.
